# Leiomyosarcoma of urinary bladder with unusual recurrence in intestinal mucosa and peritoneum: a case report

**DOI:** 10.1186/s43046-021-00095-z

**Published:** 2021-12-13

**Authors:** Poorva Vias, Shikha Goyal, Kannan Periasamy, Renu Madan, Sudheer Kumar Devana, Amanjit Bal, Reetu Kundu

**Affiliations:** 1 Department of Radiotherapy, Post Graduate Institute of Medical Education and Research, Chandigarh, 160012 India; 2 Department of Urology, Post Graduate Institute of Medical Education and Research, Chandigarh, 160012 India; 3 Department of Histopathology, Post Graduate Institute of Medical Education and Research, Chandigarh, 160012 India; 4 Department of Cytology and Gynaecological Pathology, Post Graduate Institute of Medical Education and Research, Chandigarh, 160012 India

**Keywords:** Bladder, Leiomyosarcoma, Chemotherapy, Cystectomy

## Abstract

**Background:**

Leiomyosarcomas of urinary bladder constitute rare malignant sarcomas with very few cases reported in literature.

**Case presentation:**

Here, we present a case of bladder leiomyosarcoma in a well-preserved female. She failed to respond to standard chemotherapy and had a rapidly downhill course with unusual metastases in anastomotic site and peritoneum soon after surgery. Despite multimodality management including resection of primary and metastatic site, systemic therapy and pelvic radiotherapy, our patient had dismal prognosis with an overall survival of 1.7 years.

**Conclusion:**

Leiomyosarcomas of bladder are aggressive tumors and have a very poor prognosis; thus, future research should focus on optimizing more effective treatment regimes.

## Background

Primary leiomyosarcomas of bladder are rare but aggressive tumors with a median age of 52 years and a slight male preponderance. Nearly 210 cases have been reported in adults [1]. Most (75%) are high grade, and higher mitotic activity (≥ 5/10 HPF) correlates with higher mortality [2].

Clinical features are indistinguishable from other urothelial malignancies. It is important to establish the difference between a variant urothelial histology vis-à-vis pure leiomyosarcoma for prognostication and treatment decisions. Due to the rarity of the disease, treatment recommendations are not standardized and clinical course is not known well. We describe the case of a young lady with leiomyosarcoma bladder who had poor response to local and systemic therapies and had a fulminant disease course with atypical recurrence patterns.

## Case presentation

**Fig. 1:**
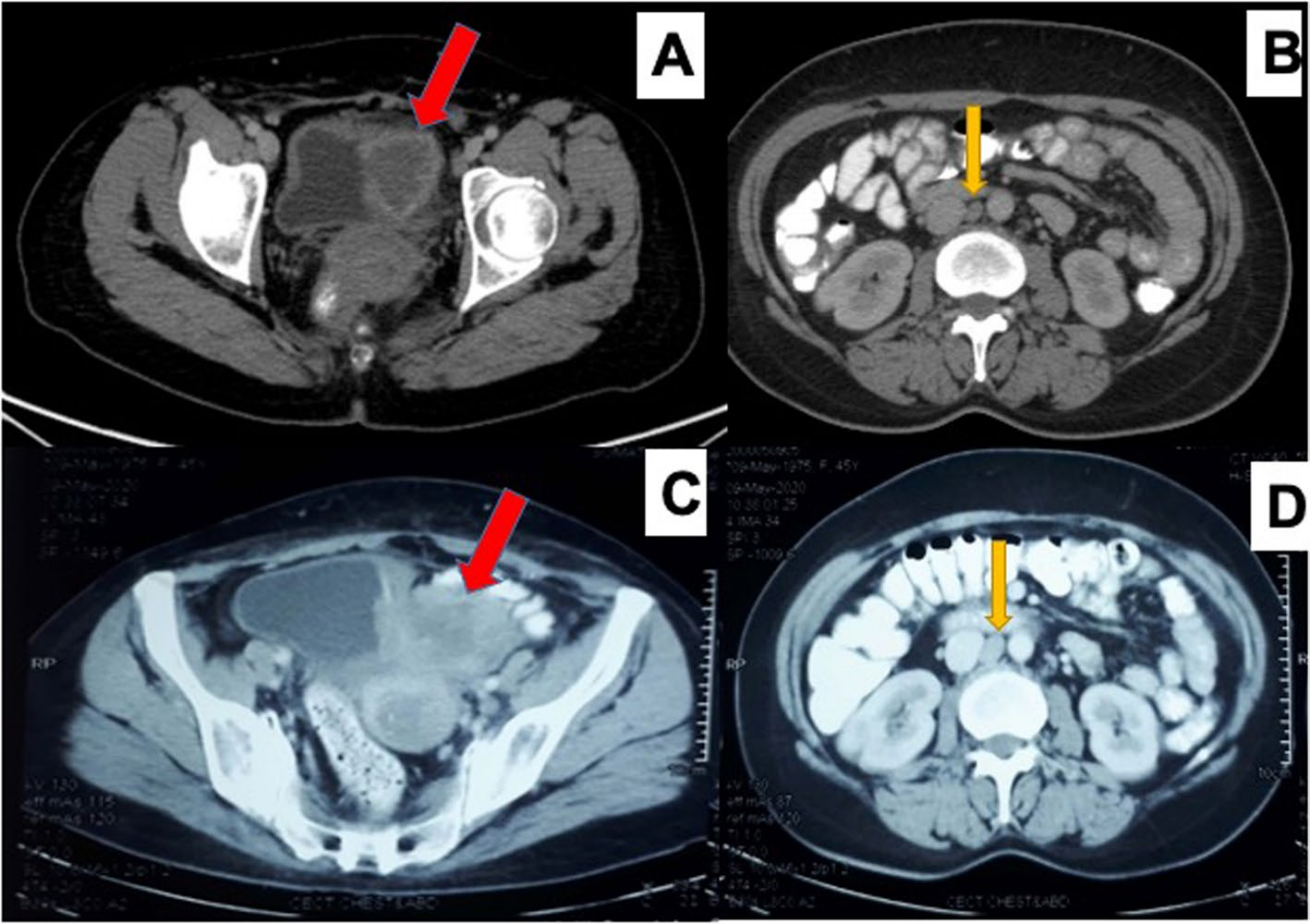
**A** Axial computed tomography (CT) scan images at baseline showing a large proliferative growth involving left lateral wall of urinary bladder with perivesical extension and vesicoureteric junction involvement (red arrow). **B** The same CT image showed multiple enlarged pelvic and paraaortic and aortocaval lymph nodes (yellow arrow) which were 18F-fluorodeoxyglucose (FDG) avid on corresponding positron emission tomography (PET) image. **C** Contrast-enhanced CT following two lines of chemotherapy showing progression in bladder growth (red arrow), while the paraaortic nodes were unchanged (yellow arrow) but still enhancing (**D**)

**Fig. 2:**
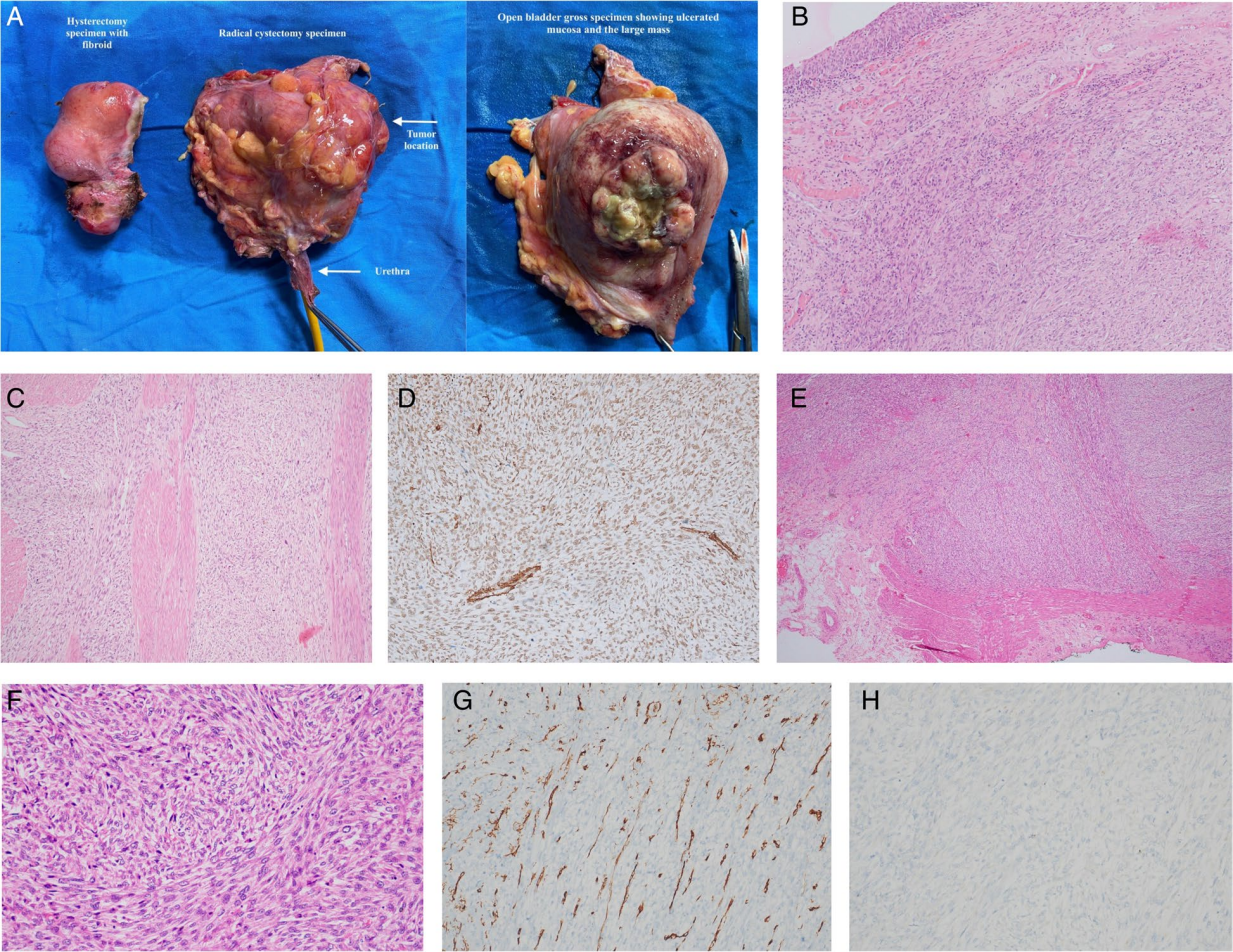
**A** Radical cystectomy specimen showing urinary bladder with a large necrotic and fungating growth arising from left lateral wall with serosal involvement. The resected hysterectomy specimen shows a leiomyoma. **B** Photomicrograph showing tumor with overlying urothelial epithelium. Eosinophilic spindle shaped tumor cells are arranged in storiform pattern and interlacing fascicles (H&E, × 40). **C** Tumor is infiltrating bladder muscle (H&E, × 40). **D** Immunohistochemistry (IHC) shows expression of smooth muscle actin (SMA) in the tumor cells (SMA immunostain, × 200). **E** Sections from intestinal mass showed an invasive well-demarcated tumor arising from muscularis mucosae and extending transmurally to reach the serosa. Photomicrograph shows spindle cell tumor arranged in short and long fascicles (H&E, × 40). **F** High magnification showing increased mitotic activity in the spindle shaped tumor cells (H&E, × 400). **G** On IHC, the tumor cells were negative for CD34, and **H** negative for DOG1

**Fig. 3:**
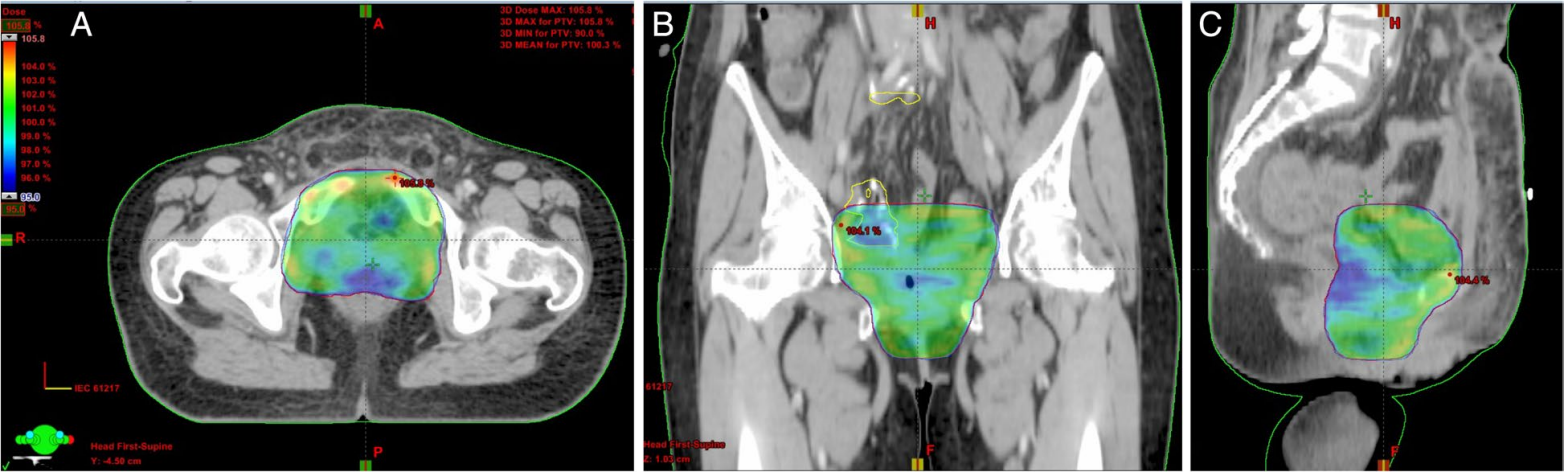
Clinical target volume (tumor bed) was delineated as per NRG guidelines for adjuvant radiotherapy for bladder cancers, and an expansion margin of 1 cm given around this volume to generate planning target volume (PTV). A dose of 50.4 Gy in 28 fractions was delivered to the PTV. Volumetric modulated radiotherapy plan of the patient, showing **A** axial, **B** coronal, and **C** sagittal views

**Fig. 4:**
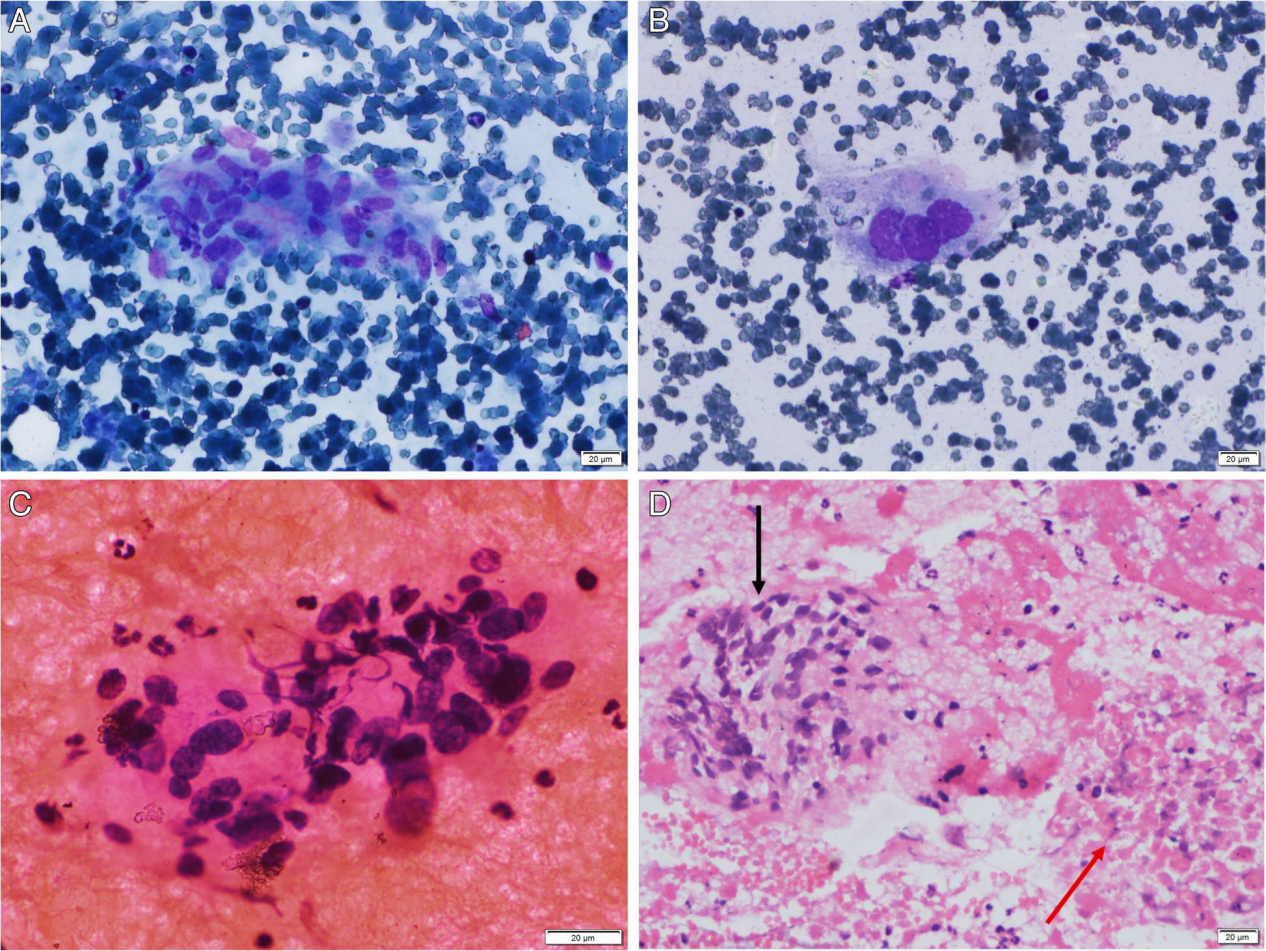
Fine needle aspiration cytology from peritoneal deposit. **A** Smear showing cluster of oval to spindled tumor cells (May-Grünwald Giemsa, MGG). **B** Aspirate smear showing a tumor giant cell (MGG). **C** Cluster of tumor cells with coarse nuclear chromatin (H&E). **D** Cell block showing fragment of viable (black arrow) and necrosed (red arrow) tumor tissue (H&E)

**Fig. 5:**
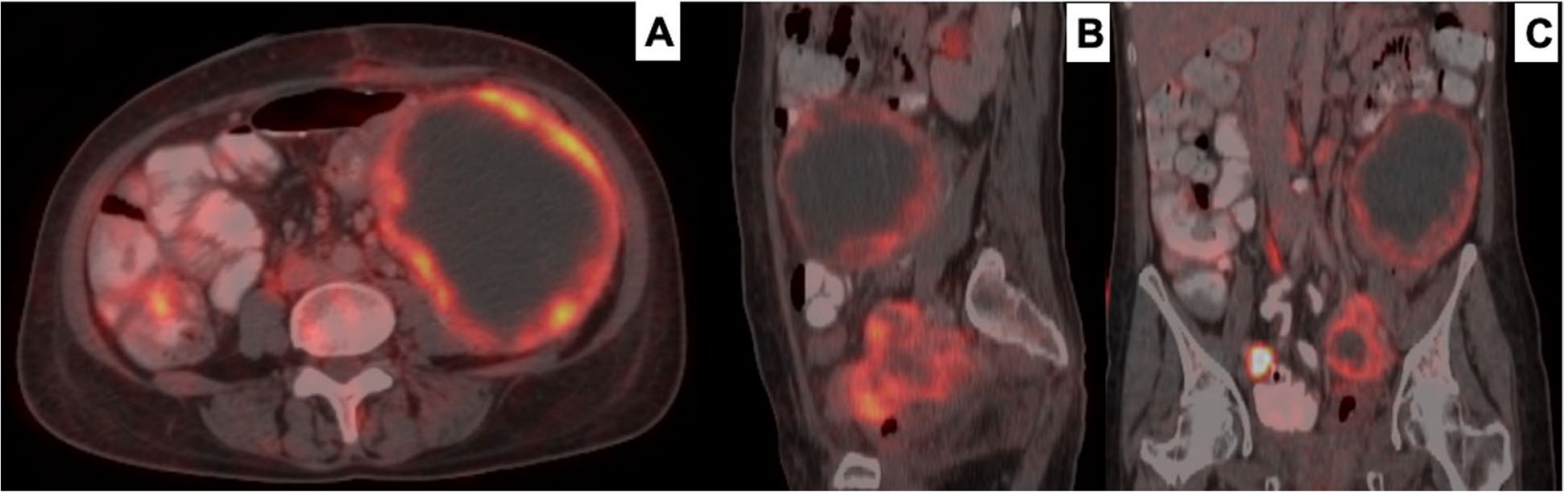
Whole body 18F-fluorodeoxyglucose (FDG) positron emission tomography (PET)-contrast-enhanced computed tomography (CECT) of the patient 6 weeks after ileal resection for anastomotic metastases showed multiple, large, necrotic omental and serosal lesions in abdomen and pelvis regions, and pelvic lymph nodes showing central necrosis and FDG avidity in the walls, largest lesion measuring 12.5 × 11.7 × 11.5 cm (SUVmax 13.8) in left lumbar region in **A** axial, **B** sagittal, and **C** coronal views

A 45-year-old lady with good performance status (PS) and otherwise unremarkable personal and family history presented in February 2019 with lower abdominal pain and dysuria for 5 months, having failed conservative measures. Ultrasound abdomen and cystoscopy revealed a left lateral bladder wall (3 × 3 cm) submucosal growth. Transurethral resection biopsy specimen showed intact transitional lining epithelium with an invasive lobular tumor in subepithelium arranged in the form of interlacing fascicles and storiform pattern comprising of eosinophilic spindle shaped tumor cells with cigar-shaped nuclei, perinuclear vacuoles and moderate amount of eosinophilic cytoplasm. The tumor cells showed brisk mitotic activity, cytological atypia and large areas of necrosis. Immunohistochemistry (IHC) was positive for smooth muscle actin (SMA) and negative for GATA3 and cytokeratin. A diagnosis of leiomyosarcoma was given. Whole body 18F-fluorodeoxyglucose (FDG) positron emission tomography (PET)-contrast-enhanced computed tomography (CECT) showed an exophytic heterogeneously enhancing soft tissue mass with central necrosis (4.9 × 5.3 × 4.6 cm, SUVmax 15.3) arising from left lateral bladder wall and having ill-defined planes with uterus at places, multiple pelvic (left external iliac, internal iliac and common iliac, largest 1.3 × 1.1 cm, SUVmax 8.7) and paraaortic nodes (largest 0.8 cm, SUVmax 7.6) and a subcm-enhancing nodule in body of right adrenal with doubtful significance (SUVmax 4.8) (Fig. 1A, B). With a diagnosis of stage IV leiomyosarcoma of bladder, she received palliative chemotherapy with VAC/IE regime (vincristine 1.4 mg/m^2^ + adriamycin 75 mg/m^2^ + cyclophosphamide 1200 mg/m^2^ iv day 1, alternating with ifosfamide 1.8 g/m^2^ + etoposide 100 mg/m^2^ day 1–3, every 3 weeks). After 3 cycles, she had persistent symptoms and local progression on CECT, for which she received palliative radiotherapy to pelvis, 30 Gy in 10 fractions, and second line chemotherapy (gemcitabine 1000 mg/m^2 ^days 1 and 8 + docetaxel 75 mg/m^2^ day 1 every 3 weeks) for 6 cycles. She had recurrent hematuria, and imaging suggested progressive bladder growth and unchanged nodal disease (Fig. 1C, D). Following multidisciplinary clinic discussion, she was advised surgery (radical cystectomy, total abdominal hysterectomy, pelvic lymphadenectomy, paraaortic sampling) in view of good PS and persistent symptoms. Intraoperatively, a large fungating solid growth (9 × 6 cm) arising from left lateral bladder wall, and adherent to left lateral pelvic wall and an ileal loop was seen. Bilateral ureters, uterus and vaginal cuff were uninvolved. There were no peritoneal deposits or ascites. Left paraaortic and aortocaval nodes were enlarged and firm in consistency. Bilateral pelvic nodes were not enlarged. Postoperative histopathology showed a polypoidal mass of 6.5 × 6 × 5 cm with perivesical fat invasion and close circumferential margin (1 mm). Morphology showed a spindle cell tumor cells arranged in short and long fascicles, with high mitotic activity. Morphology and IHC were identical with initial biopsy. Overall picture confirmed the earlier diagnosis of pleomorphic leiomyosarcoma (Fig. 2A–D). Pelvic and paraaortic nodes showed granulomas secondary to tumor antigen. Ziehl–Neelsen staining for acid fast bacilli was negative in the lymph nodes. The initial diagnosis of metastatic disease (in paraaortic nodes) was revised since the nodes had remained unchanged during serial imaging, and she was offered adjuvant radiotherapy (50.4 Gy in 28 fractions) based on NRG guidelines, after due consideration of prior radiotherapy dose and volume [3] (Fig. 3). Two months after radiotherapy, she reported to emergency with projectile vomiting, constipation and upper gastrointestinal bleed. Acute intestinal obstruction related to treatment-related pelvic fibrosis was suspected. CECT abdomen showed jejunal and ileal dilatation with no definite growth or site of obstruction. Emergency laparotomy was done and an obstructing growth 5 × 4 cm arising from intestinal mucosa at ileal anastomotic site was seen on exploration. Resection and anastomosis were done. Histopathology of the intestinal mass showed an invasive well-demarcated tumor apparently arising from muscularis mucosae and extending transmurally to reach the serosa. There was brisk mitotic activity and microscopic foci of necrosis. No lymphovascular or perineural invasion were identified. Resection margins and mesenteric margins are free. Four lymph nodes were identified that showed reactive changes. IHC was positive for SMA and negative for CD34, DOG1 and c-kit, ruling out the possibility of gastrointestinal stromal tumor. The histopathologic picture suggested metastatic leiomyosarcoma (Fig. 2E–H). Within 6 weeks following surgery, she again presented to emergency with a progressive intraabdominal (left lumbar) soft tissue swelling, initially suspected as an abscess based on ultrasound, but subsequently proven metastatic on cytology (Fig. 4). PET-CECT showed multiple paraaortic nodes (largest 1.6 cm, SUVmax 5.7) and serosal deposits (largest 7.3 × 4.6 cm, SUVmax 13.8) in pelvis with central necrosis and ill-defined fat planes with sigmoid colon, ileal loops, and left external iliac vessels (Fig. 5). Due to low PS, she was advised metronomic chemotherapy [4]. However, she could not start the same due to declining oral intake and succumbed to her illness 2 months after last PET imaging, with an overall survival of 1.7 years since diagnosis.

## Discussion

Only 5% bladder malignancies have non-urothelial origin; of these, leiomyosarcomas constitute < 0.1% [5]. The most common complaint is painless hematuria, with dysuria/abdominal pain similar to our case reported in < 20%. Our patient did not have any prior radiation or chemotherapy (cyclophosphamide) exposure to explain the disease origin. Lymph nodal involvement at presentation is rare (< 3%). Granulomas in draining lymph nodes of tumors have occasionally been noted even in the absence of malignant cells in these nodes, with an uncertain prognostic value. Earlier hypotheses have included T cell-mediated immune response to soluble antigens released by tumors or persistence of non-degradable products leading to granulomatous inflammation [6]. Every effort should be made to rule out even a small focus of tumor within such granulomatous nodes. Leiomyosarcomas are not known to have high propensity for lymph node metastases, unlike rhabdomyosarcomas, epithelioid sarcomas, clear cell sarcomas, angiosarcomas, or synovial sarcomas, and any formal studies quantifying the false positive rates of nodal detection on PET imaging in sarcomas are lacking [7]. Inflammatory conditions including sarcoidosis and infections, especially tuberculosis in endemic countries, are common mimics of malignancies due to high FDG avidity, leading to false-positive results and should be ruled out [8]. We did not perform upfront nodal biopsy to confirm malignancy in PET-positive nodes in our patient. In view of little change in size/appearance with chemotherapy and histopathological determination of granulomatous change post-surgery, we revised our initial staging and offered her aggressive local adjuvant therapy for close margins and perivesical extension.

Treatment for bladder leiomyosarcomas is individualized based on tumor size and disease extent. Radical cystectomy is the most commonly described treatment (43%), and neoadjuvant or adjuvant chemotherapy/radiotherapy has been employed in a quarter of cases [1, 9]. Adjuvant/neoadjuvant therapy doubles disease-specific survival. Combination chemotherapy including ifosfamide and anthracyclines is most commonly used, akin to other soft tissue sarcomas, and taxane/gemcitabine based regimes are employed in second line. In our patient, both these regimes failed to elicit symptomatic relief or tumor regression. Surgical margin status is prognostic and patients with positive/close margins should be offered adjuvant radiotherapy. MD Anderson data on 36 bladder leiomyosarcomas treated between 1996 and 1998 suggested the prognostic importance of MSKCC staging incorporating size, grade, and depth of invasion [10]. Another series from the same institution on 14 bladder leiomyosarcomas showed that under 10% received adjuvant radiotherapy [11]. Nearly 25% patients recur, most within the pelvis and within 3 years of diagnosis. Pelvic recurrences are most commonly seen in the postoperative bed. Risk of distant relapse remains high, with systemic disease (lung, bone, liver, brain) developing in nearly half the patients [10]. For urothelial malignancies treated with radical cystectomy and urinary diversion, recurrences within ileal conduit and upper urothelial tract are occasionally reported [12]. However, intestinal mucosal wall metastases or peritoneal metastases from either urothelial malignancies or bladder sarcomas have not been reported earlier. Metastases to anastomotic site or to peritoneum may theoretically arise from surgical spillage and contamination of anastomosis through iatrogenic implantation of tumor cells [13]. In our patient, surgery on both occasions was done by oncosurgeons following oncologic principles and no tumor rupture or intraoperative spill were noted. However, the possibility of contamination during specimen retrieval cannot be ruled out conclusively. The histopathologic appearance of the tumor on all three instances (biopsy, cystectomy and bowel resection) was aggressive with necrotic change indicating an aggressive biology that was poorly responsive to all adjuvant therapies (local, systemic) and this was possibly the more probable cause of the rapid fulminant course.

Disease-specific mortality (5 years) is reported at 38% overall and 58% for high-grade leiomyosarcomas. Survival outcomes of leiomyosarcomas appear similar to other sarcomas in MD Anderson series.

## Conclusion

Our patient represents presentation with atypical symptoms, inadequate response to systemic therapies and unusual metastases in intestinal mucosa and peritoneum, and adds to scant available literature on presentation and relapse patterns of bladder leiomyosarcomas. Sampling of pelvic nodes upfront (and determination of non-involvement by malignancy) would possibly have enabled early surgery and tailored adjuvant therapies, possibly improving her outcome. Following histopathologic diagnosis of pure bladder sarcomas, future protocols could involve biopsy confirmation of any unusual sites (e.g., enlarged or FDG avid lymph nodes, mucosal, or serosal deposits) and multidisciplinary tumor board (MDT)-based treatment decisions to offer the best quality of care. Recurrences are early and responses to neoadjuvant systemic therapy are heterogeneous; hence in localised disease, efforts should be made for early surgery and tailored adjuvant therapy.

## Data Availability

Data can be made available by authors on reasonable request.

## Abbreviations

VAC/IE: Vincristine, adriamycin, cyclophosphamide, ifosfamide, etoposide
CECT: Contrast-enhanced computed tomography
FDG: Fluorodeoxyglucose
H&E: Hematoxylin and eosin
IHC: Immunohistochemistry
MDT: Multidisciplinary tumor board
MSKCC: Memorial Sloan Kettering Cancer Center
PET: Positron emission tomography
PS: Performance status
SMA: Smooth muscle actin

## References

[1] Zieschang H, Koch R, Wirth MP, Froehner M. Leiomyosarcoma of the urinary bladder in adult patients: a systematic review of the literature and meta-analysis. Urol Int. 2019;102(1):96–101. 10.1159/000494357. 30384363 10.1159/000494357

[2] Lee TK, Miyamoto H, Osunkoya AO, Guo CC, Weiss SW, Epstein JI. Smooth muscle neoplasms of the urinary bladder: a clinicopathologic study of 51 cases. Am J SurgPathol. 2010;34(4):502–9. 10.1097/PAS.0b013e3181cf326d. 10.1097/PAS.0b013e3181cf326d 20154594

[3] Baumann BC, Bosch WR, Bahl A, Birtle AJ, Breau RH, Challapalli A, et al. Development and validation of consensus contouring guidelines for adjuvant radiation therapy for bladder cancer after radical cystectomy. Int J Radiat Oncol Biol Phys. 2016;96(1):78–86. 10.1016/j.ijrobp.2016.04.032 Epub 2016 May 7. Erratum in: Int J Radiat Oncol Biol Phys. 2016;96(5):1129. 27511849 10.1016/j.ijrobp.2016.04.032 PMC5207044

[4] Pramanik R, Agarwala S, Gupta YK, Thulkar S, Vishnubhatla S, Batra A, et al. Metronomic chemotherapy vs best supportive care in progressive pediatric solid malignant tumors: a randomized clinical trial. JAMA Oncol. 2017;3(9):1222–7. 10.1001/jamaoncol.2017.0324. 28384657 10.1001/jamaoncol.2017.0324 PMC5824286

[5] Hamadalla NY, Rifat UN, Safi KC, Mohammed M, Abu-Farsakh H. Leiomyosarcoma of the urinary bladder: a review and a report of two further cases. Arab J Urol. 2013;11(2):159–64. 26558075 10.1016/j.aju.2013.03.004 PMC4443010

[6] Bhatia A, Kumar Y, Kathpalia AS. Granulomatous inflammation in lymph nodes draining cancer: a coincidence or a significant association! Int J Med Med Sci. 2009;1(2):13–6.

[7] Benz MR, Crompton JG, Harder D. PET/CT variants and pitfalls in bone and soft tissue sarcoma. Semin Nucl Med. 2021;S0001-2998(21):00041–6. 10.1053/j.semnuclmed.2021.06.009 34238508

[8] Sümbül AT, Sezer A, Abali H, Gültepe B, Koçer E, Reyhan M, et al. An old enemy not to be forgotten during PET CT scanning of cancer patients: tuberculosis. Contemp Oncol (Pozn). 2016;20(2):188–91. 27358601 10.5114/wo.2014.43985 PMC4925724

[9] Venyo AK. Leiomyosarcoma of the urinary bladder: a review and update of the literature. J Cancer Res Oncobiol. 2019;2(2):126.

[10] Rosser CJ, Slaton JW, Izawa JI, Levy LB, Dinney CP. Clinical presentation and outcome of high-grade urinary bladder leiomyosarcoma in adults. Urology. 2003;61:1151–5. 10.1016/s0090-4295(03)00021-9. 12809885 10.1016/s0090-4295(03)00021-9

[11] Spiess PE, Kassouf W, Steinberg JR, Tuziak T, Hernandez M, Tibbs RF, et al. Review of the M.D. Anderson experience in the treatment of bladder sarcoma. Urol Oncol. 2007;25(1):38–45. 10.1016/j.urolonc.2006.02.003. 17208137 10.1016/j.urolonc.2006.02.003

[12] Eloubeidi MA, Varadarajulu S, El-Galley R, Bueschen AJ, Eltoum I. EUS-guided FNA for the diagnosis of recurrent bladder cancer through the ileal conduit: a novel approach. Gastrointest Endosc. 2006;64(3):450–3. 10.1016/j.gie.2006.02.050. 16923503 10.1016/j.gie.2006.02.050

[13] Gresham E, Don PF. Iatrogenic implantation of cancer cells during surgery. Hawaii J Health Soc Welf. 2020;79(1):4–6. 31967105 PMC6969391

